# Association between medication burden and acute care use in older metastatic prostate cancer patients on androgen receptor signaling inhibitors

**DOI:** 10.1002/cncr.70163

**Published:** 2025-11-02

**Authors:** Michael A. Liu, Rohit Raghunathan, Karie Runcie, Margaux Wooster, Shikun Wang, Jason D. Wright, Alexander Z. Wei, Mark Stein, Dawn L. Hershman

**Affiliations:** ^1^ Herbert Irving Comprehensive Cancer Center Columbia University Irving Medical Center New York New York USA; ^2^ Feinberg School of Medicine Robert H. Lurie Comprehensive Cancer Center Northwestern University Chicago Illinois USA

**Keywords:** acute care use, androgen receptor signaling inhibitors, medication adherence, older adults, polypharmacy, prostate cancer

## Abstract

**Background:**

Management of metastatic prostate cancer often requires combining androgen deprivation therapy (ADT) with novel androgen receptor signaling inhibitors (ARSIs). Although these agents improve survival, older patients may face acute care utilization from medication burden, reflected in polypharmacy and nonadherence.

**Methods:**

Using SEER‐Medicare data, the authors identified patients ≥66 years old with de novo metastatic prostate cancer prescribed abiraterone, enzalutamide, or apalutamide (2010–2017). Polypharmacy was defined by the Youden index (≥8 medications). ARSI adherence was measured by medication possession ratio (≥0.8) from initiation to discontinuation, assessed over 6 months. Acute care use was defined as any inpatient hospitalization or emergency visit within 6 months. Demographic characteristics were compared by *t*‐tests/χ^2^. Negative binomial regression estimated incidence rate ratios (IRRs) for acute care use.

**Results:**

Among 2697 patients (mean age, 75 years), most were White (80.3%), married (63.1%), and received prior ADT (85.3%). Polypharmacy was present in 50.6% of patients before ARSI initiation, whereas ARSI nonadherence in the 6 months post‐initiation was 34.0%. Polypharmacy and adherence were not significantly associated. In adjusted analyses controlling for demographic, clinical, and treatment factors, both polypharmacy (IRR, 1.59; 95% confidence interval [CI], 1.28–1.98) and ARSI nonadherence (IRR 2.50; 95% CI, 2.00–3.03) independently prognosticated higher acute care use.

**Conclusions:**

Medication burden, as characterized by suboptimal adherence and polypharmacy, is an independent risk factor for acute care use among older adults initiating ARSI treatment for metastatic prostate cancer. These findings highlight an opportunity for potential interventions to reduce downstream acute care use.

## Abstract 2

In this Surveillance, Epidemiology, and End Results‐Medicare based study of older adults with metastatic prostate cancer, both polypharmacy and nonadherence to androgen receptor signaling inhibitors were common and independently associated with increased acute care use. These results highlight the importance of addressing medication burden to improve outcomes in this high‐risk population.

## INTRODUCTION

Metastatic prostate cancer is a leading cause of cancer‐related death in men. Treatments can be complex involving multiple oral medications. Optimal management of metastatic prostate cancer may involve the combination of androgen deprivation therapy (ADT) with novel oral androgen receptor signaling inhibitors (ARSIs) such as abiraterone, enzalutamide, and apalutamide. Although these newer agents have revolutionized care and improved survival, older patients may be at risk for negative outcomes from polypharmacy or low medication adherence, due to the complexity of the treatments.

Older adults are particularly susceptible to polypharmacy due to factors such as age‐related changes in pharmacokinetics and pharmacodynamics, increased comorbidities, cognitive decline, and limited data on medication safety and efficacy in this population.^1^, ^2^ Polypharmacy has a known high prevalence in older adults with cancer, and nearly all patients taking oral cancer‐directed therapies are on five or more medications.^3^ A meta‐analysis evaluating for outcomes in polypharmacy defined at this threshold demonstrated mixed results with several studies suggesting increased risk of treatment toxicity, falls, and medication nonadherence, but the association with survival was minimal and none showed an association with chemotherapy completion.^4^ A follow‐up study found that a cutoff of eight medications was more sensitive, and polypharmacy to this degree (but not with five medications) was associated with functional impairments and negative outcomes.^5^ Additionally, polypharmacy in patients with prostate cancer who were receiving intravenous chemotherapy has been shown to lead to increased acute care use in a prior Surveillance Epidemiology and End Results (SEER)‐Medicare study.^6^

Prior studies have shown a close link between polypharmacy and medication adherence, with increased prevalence in patients with chronic conditions.^7^ As polypharmacy rises alongside life expectancy from expanded treatment options,^8^ medication adherence becomes an increasing priority in chronic cancer management. Poor adherence is associated with worsened disease, increased health care costs, and death, with the most significant drop‐off in adherence after the first 6 months of therapy.^7^, ^9^ The majority of studies of medication adherence in older patients with cancer have been among patients with breast cancer, but data are limited on patients with prostate cancer.^10^

Given the demonstrated importance of medication burden, we hypothesized that polypharmacy and ARSI adherence are prevalent and may contribute to increased acute care use in older patients with metastatic prostate cancer.

## MATERIALS AND METHODS

### Study population

This was a retrospective study using the SEER‐Medicare database. SEER data are derived from population‐based cancer registries representing more than 30% of the US population. Medicare data include demographic and vital status information on all Medicare beneficiaries along with administrative claims on health care services provided in the inpatient and outpatient settings including skilled nursing care and cancer therapies. Medicare Part D data files include additional oral prescription drug information.

**FIGURE 1:**
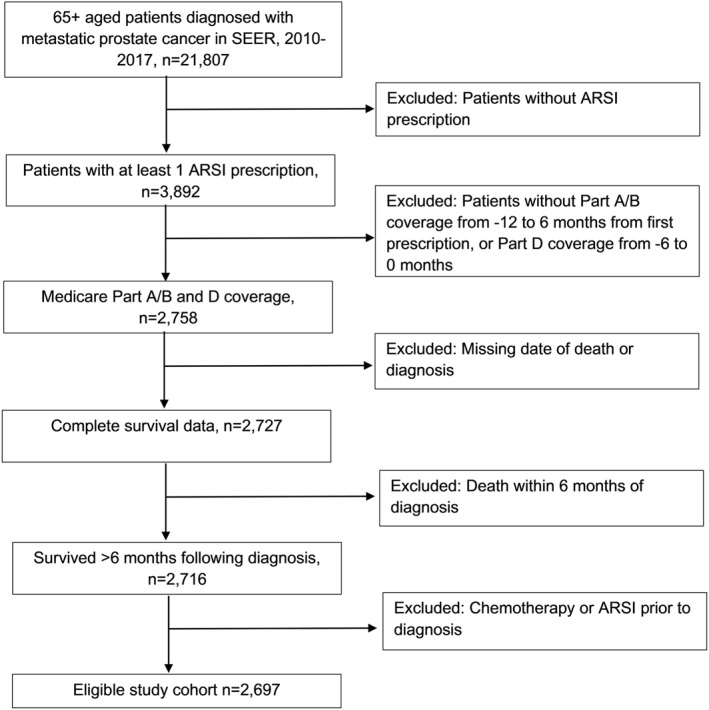
Flow chart of cohort.

The study population consisted of patients aged 66 and older with de novo metastatic prostate cancer who were prescribed abiraterone, enzalutamide, or apalutamide from 2010 to 2017, defined by Medicare Part D claims data. Eligible patients had continuous Medicare Part A/B coverage 12 months before and 6 months after drug initiation, and Part D coverage 6 months before initiation. The start date of 2010 corresponds to 1 year before the approval of abiraterone in the metastatic prostate setting. Patients were excluded if the date of death was within 6 months of diagnosis, date of death was unknown, or if chemotherapy was administered before ARSI. A flow diagram of the cohort selection process is illustrated in Figure 1, which was replicated from a prior manuscript.^11^

### Definitions

ARSIs including abiraterone, enzalutamide, and apalutamide were identified using International Classification of Diseases codes for treatments. These medications were selected as the focus because they represented the newly approved oral medications during the study time frame, with requirements of long‐term adherence. Patients were also evaluated for ADT treatments received before ARSI. This list consisted of leuprolide, bicalutamide, degarelix, triptorelin, flutamide, nilutamide, goserelin, and histrelin.

Age, race, marital status, and region were obtained from SEER cancer files. Age was measured as a continuous variable. Race was classified as White, Black, or other. Marital status was categorized as married, unmarried, or unknown. Region was grouped into East, Northern Plains, Pacific Coast, and Southwest. Socioeconomic status was approximated based on regional high school education level (non–high school >10% or non–high school <10%).

Polypharmacy was defined based on the Youden index with a cutoff of eight or more medications, using the 6‐month window before ARSI initation.^12^ We selected this cutoff based on a recent study on older patients with advanced tumors that found this to be the optimal cutoff for polypharmacy in predicting outcomes.^12^ Medication adherence for anti‐androgens was measured using the medication possession ratio (MPR). MPR is calculated as the sum of days’ supply for all medication fills divided by the days between the first and last fills plus the days’ supply of the last fill. An MPR ≥0.8 defined adherence, which is consistent with prior studies.^13^ Adherence to anti‐androgens was measured from the first to the last use at starting ARSI treatment, in a 6‐month interval. Acute care use was defined as any inpatient hospitalization or emergency visit event within 6 months after initiation of oral anti‐androgen therapies. This time frame was selected due to the known risk interval of increased adverse events after starting ADT.^14^

### Statistics

Demographic variables were reported via means and standard deviations for continuous variables and proportions for categorical variables. *t*‐Test and χ^2^ were used to compare demographic characteristics between patients with and without acute care use, polypharmacy, and ARSI adherence. Multivariable analysis was performed using negative binomial regression to calculate incidence rate ratios of acute care events alongside 95% confidence intervals (CIs), comparing the total number of admissions to the total time at risk. An α level of 0.05 was used as the cutoff to denote significance.

## RESULTS

A total of 2697 patients with metastatic prostate cancer on ARSIs were included in the analysis (Table 1). The average age of the cohort was 75 with a standard deviation of 7 years, and most patients were White (80.3%) and married (63.1%).

**TABLE 1:** Baseline cohort characteristics % (*n*).

	Polypharmacy				ARSI Adherence		*p*
	All (*n* = 2697)	Yes (*n* = 1365)	No (*n* = 1332)	*p*	Yes (*n* = 1780)	No (*n* = 917)	
NCI index (mean ± SD)	0.39 ± 0.53	0.43 ± 0.56	0.18 ± 0.35	<.001**	0.31 ± 0.48	0.33 ± 0.51	.37
Age, years	75.0 ± 7.0	75.2 ± 6.7	74.9 ± 7.0	.29	74.9 ± 6.9	75.2.±7.1	.32
Race							
White	80.3 (2165)	81.3 (1110)	79.2 (1055)	.34	79.9 (1422)	81.0 (743)	.76
Black	12.1 (326)	11.7 (159)	8.3 (110)		12.3 (218)	11.8 (108)	
Other	7.6 (205)	7.0 (96)	12.5 (167)		7.9 (140)	7.2 (206)	
Marital status							
Married	63.1 (1703)	66.1 (902)	60.1 (801)	.0036**	64.1 (1141)	61.3 (562)	.32
Unmarried	29.3 (789)	27.4 (374)	31.2 (415)		28.7 (510)	30.4 (279)	
Unknown	7.6 (205)	6.5 (89)	8.7 (116)		7.3 (120)	8.3 (76)	
Region							
East	39.3 (1061)	42.4 (579)	36.2 (482)	.0056**	39.8 (709)	38.4 (352)	.0092**
Northern Plains	10.9 (294)	10.6 (145)	11.2 (149)		12.1 (215)	8.6 (79)	
Pacific Coast	44.9 (1211)	41.9 (572)	48.0 (639)		43.8 (779)	47.1 (432)	
Southwest	4.9 (131)	5.1 (69)	4.7 (62)		4.3 (77)	5.9 (54)	
Socioeconomic status							
Non–high school >10%	50.5 (1358)	50.6 (689)	50.3 (669)	.91	50.3 (894)	50.8 (464)	.81
Non–high school <10%	49.6 (1334)	49.4 (674)	49.7 (660)		49.7 (884)	49.2 (450)	
ARSI treatments							
Abiraterone	76.2 (2056)	76.8 (1048)	75.7 (1008)	.50	78.5 (1397)	71.9 (659)	<.001**
Enzalutamide or apalutamide	71.8 (1938)	71.5 (976)	72.1 (962)	.82	67.1 (539)	74.8 (1328)	<.001**
Prior ADT							
ADT before ARSI	85.3 (2300)	88.1 (1202)	82.4 (1098)	<.001**	86.6 (1541)	82.8 (759)	.0083**
No ADT before ARSI	14.7 (397)	11.9 (163)	17.6 (234)		13.4 (239)	17.2 (158)	

Abbreviations: ADT, androgen deprivation therapy; ARSI, androgen receptor signaling inhibitor; NCI, National Cancer Institute.

**p < .01.

### Polypharmacy

**FIGURE 2:**
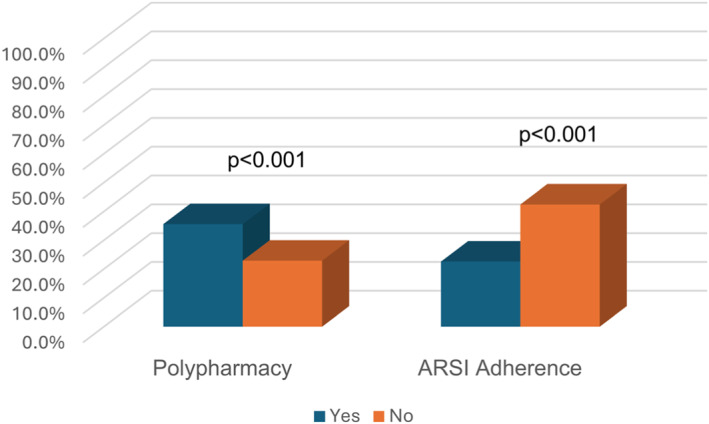
Acute care use by polypharmacy status and androgen receptor signaling inhibitor adherence.

Polypharmacy was present in slightly above half (50.6%) of patients before initiation of ARSI therapy (Figure 2). Patients with polypharmacy were more likely to have a higher mean NCI comorbidity index (0.43 vs. 0.18, *p* < .001), be married (66.1% vs. 60.1%, *p* = .006), live in the East (42.4% vs. 36.2%, *p* = .0056), and have received prior ADT (88.1% vs. 82.4%, *p* < .001). There were no significant associations of age, race, socioeconomic status, or ARSI treatment with polypharmacy.

### ARSI adherence

ARSI nonadherence in the 6 months post‐initiation was 34.0%. There was no significant association of polypharmacy with ARSI adherence; those with polypharmacy had a nonadherence rate of 34.9% compared to 33.1% in those without polypharmacy (*p* = .34). Patients with ARSI nonadherence were less likely to have received abiraterone (71.9% vs. 78.5%, *p* < .001) compared to enzalutamide (74.3% vs. 66.3%, *p* < .001) and less likely to receive ADT before ARSI (82.8% vs. 86.6%, *p* = .0083). There were no significant relationships of age, race, marital status, socioeconomic status, or NCI comorbidity index with ARSI adherence.

### Acute care use

Acute care use within 6 months after ARSI initiation was present in 29.5% of patients overall. Patients with acute care use were significantly more likely to: be older (mean age, 75.7 vs. 74.6 years; *p* < .001), have a higher NCI comorbidity index (mean, 0.43 vs. 0.26; *p* < .001), live in the Eastern United States (42.0% vs. 38.3%; *p* = .03), and not have received enzalutamide (66.3% vs. 73.1%; *p* < .001) or apalutamide (0.13 vs. 1.0%; *p* = .02). Most patients received ADT before ARSI (85.3%), and there was no significant difference in acute care use among those who received ADT compared to those who did not. Polypharmacy was associated with higher acute care use (35.6% vs. 22.9%; *p* < .001). ARSI nonadherence was also significantly associated with acute care use (42.4% vs. 22.6%; *p* < .001).

### Multivariable analysis

In a multivariable analysis adjusting for demographic, clinical, and treatment‐related covariates (Table 2), nonadherence to ARSIs was independently associated with more than a 2‐fold increased incidence of acute care events (incidence rate ratio [IRR], 2.50; 95% CI, 2.00–3.03). Similarly, polypharmacy was also associated with an increased incidence rate of acute care use (IRR, 1.59; 95% CI, 1.28–1.98). Advanced age and increased comorbidity index were also associated with increased acute care use (Table 2). There was no association of race, marital status, region, or socioeconomic status and acute care use.

**TABLE 2:** Multivariable regression: Medication variables with acute care use.

Covariates	IRR (95% CI)	*p*
Medication variables		
ARSI non‐adherence (yes vs. no)	2.50 (2.00–3.03)	<.001**
Polypharmacy (yes vs. no)	1.59 (1.28–1.98)	<.001**
Drug type (abi vs. enza/apa)	0.96 (0.74–1.24)	.06
Demographics		
Age (per year)	1.02 (1.00–1.03)	.01*
Race (Black vs. White)	0.98 (0.70–1.37)	.88
Race (other vs. White)	1.00 (0.65–1.53)	.99
Marital status (unmarried vs. married)	1.13 (0.90–1.42)	.28
Region (Northern Plains vs. East)	1.01 (0.73–1.40)	.95
Region (Pacific Coast vs. East)	0.95 (0.75–1.20)	.65
Region (Southwest vs. East)	1.23 (0.78–1.95)	.37
SES (non–high school >10% vs. <10%)	1.01 (0.82–1.24)	.96
Comorbidities		
NCI comorbidity index (per unit)	1.79 (1.45–2.21)	<.001**

Abbreviations: abi, abiraterone; apa, apalutamid; ARSI, androgen receptor signaling inhibitor; CI, confidence interval; enza, enzalutamide; IRR, incidence rate ratio; NCI, National Cancer Institute; SES, socioeconomic status.

*p < .05, **p < .01.

## DISCUSSION

Minimizing medication burden is crucial in older adults with metastatic prostate cancer, a population that has frequent exposure to complex treatment regimens that lead to risk of adverse health events. In this cohort of older metastatic prostate cancer patients on ARSIs, ARSI nonadherence was common and independently associated with over a 2‐fold increased risk of acute care use. Additionally, over half of patients had evidence of polypharmacy, which was associated with an increased risk of acute care use.

Using a cutoff of eight or more medications, over half of the patients in this cohort experienced polypharmacy, which was slightly higher than the 43% observed in the literature.^5^ This may reflect the unique burden and comorbidities in older patients with prostate cancer who must often manage various comorbidities alongside their cancer diagnosis. Polypharmacy in older adults with prostate cancer has been associated with decreased physical function as well as health‐related quality of life.^5^, ^15^ Our study corroborates these findings by demonstrating a strong association of polypharmacy with acute care use. Potential reasons may include interference with metabolism of medications, medication nonadherence for chronic conditions, or more toxicities, which may also confound clinical assessments. These cumulative impacts of polypharmacy may lead to delays in appropriate interventions and more severe or prolonged hospitalizations.^16^ This highlights the importance of considering early preventative approaches and medication reconciliation in this higher risk population.

The ARSI adherence rate in this study was lower than that reported by other studies that used the MPR definition, where investigators found rates to be approximately 85%–92% among metastatic prostate cancer patients.^17^, ^18^ Likely, the selection of older patients in this study was a significant contributor to this difference. A systematic review focused on real‐world patients with prostate cancer found that nonadherence rates of oral medications may be between 25% and 50% in older patients with prostate cancer.^19^ Reported adherence rates for oral antineoplastic medications can vary significantly based on patient population, study setting, medication type, method of assessment and adherence calculation, and follow‐up duration.^20^ Lack of adherence to cancer medications in older adults can be related to side effects, treatment regimen complexity, mental health issues, cost or financial barriers, or lack of perceived benefit.^18^ We found in our prior study among patients with solid tumors that 13% of new oral anticancer drugs were never received, most commonly due to a change in patient or clinical decision‐making, and second due to financial access issues.^21^ Further work is needed to investigate the reasons for nonadherence among patients with metastatic prostate cancer. Addressing the barriers to ARSI adherence through patient education, treatment plan consolidation, and improved communication can play an important role in improving outcomes.

The temporal association between ARSI nonadherence and acute care utilization can be challenging to assess through an observational study. Acute care utilization itself may lead to temporary discontinuation of medications or reflect underlying disease progression and frailty not measured in claims data. However, the causal pathway that links ARSI nonadherence to acute care use is plausible. Suboptimal androgen blockade may accelerate prostate cancer disease progression, worsen symptoms, increase metabolic complications, and contribute to preventable hospitalizations. Nonadherence to chronic medications has been shown to predict ARSI nonadherence, further supporting the link between medication burden, adherence, and outcomes in this population.^22^

Interestingly, we found no significant associations between demographic variables including race, marital status, or socioeconomic status and acute care use. This should not be interpreted as proof that disparities do not exist in patients with prostate cancer. For example, the relatively small percentage of African American patients in our cohort (∼12%) limits the power to detect racial differences. Moreover, claims‐based SEER‐Medicare data may not be optimal for capturing the effects of social determinants of health, health literary, or other structural inequalities that may contribute to health disparities. Thus, database limitations may be responsible for these findings rather than a true absence in disparities.

This study has several additional limitations. Its retrospective observational design means that a causal relationship between adherence and polypharmacy with acute care use cannot be confirmed. Residual confounding and selection bias are important to consider, especially in the older adult population. SEER‐Medicare does not capture caregiver support, frailty, cancer severity (high vs. low volume, end‐organ damage), and functional status, all of which may affect medication adherence and acute care use. Additionally, selection bias may lead toward a healthier cohort with longer survival, as patients needed to have continuous Medicare coverage to be eligible, disproportionately excluding frailer or lower income patients. Confounding by indication may be present if patients with lower adherence to medications differ in ways not captured by the data set, such as lower cognitive function or levels of social support. In addition, the use of MPR to assess adherence is an estimate based on pharmacy refill data but does not directly confirm patients’ actual medication usage, short‐term treatment interruptions, or reasons for nonadherence. Additionally, we were unable to stratify by therapeutic class, which may be clinically meaningful; for example, known interactions between ARSIs and cardiac medications (e.g., direct oral anticoagulants, statins) could exacerbate adverse events and drive acute care utilization. Furthermore, we were unable to distinguish “appropriate” polypharmacy to manage multiple comorbidities from “potentially inappropriate” polypharmacy where patients are on medications for unclear purposes. Finally, data were only available from 2010 to 2017, and treatment of metastatic prostate cancer has evolved since then, with the ARSI treatments now being used in the first‐line setting, and sometimes alongside chemotherapy through a “triple therapy” regimen.

Our study underscores the importance of both adherence to ARSI treatments and addressing polypharmacy in the management of older adults with metastatic prostate cancer, with both factors contributing independently to acute care use. Improving medication adherence through education, access, and care coordination can enhance outcomes in the older adult population. Additionally, addressing polypharmacy alongside the initiation of prostate cancer directed therapy might prevent downstream acute care use. Ultimately, this study highlights a need and opportunity for more individualized and interdisciplinary care in managing medication burden in older patients with metastatic prostate cancer.

## AUTHOR CONTRIBUTIONS

**Michael A. Liu:** Conceptualization; writing–original draft; writing–review and editing; formal analysis. **Rohit Raghunathan:** Formal analysis; writing–review and editing. **Karie Runcie:** Writing–review and editing. **Margaux Wooster:** Writing–review and editing. **Shikun Wang:** Formal analysis; writing–review and editing. **Jason D. Wright:** Writing–review and editing. **Alexander Z. Wei:** Writing–review and editing. **Mark Stein:** Writing–review and editing. **Dawn L. Hershman:** Conceptualization; writing–review and editing; supervision.

## CONFLICT OF INTEREST STATEMENT

Mark Stein reports consulting fees from Johnson and Johnson International. Jason D. Wright reports consulting fees from the American College of Obstetricians and Gynecologists and Wolters Klewer Health, Inc; and grant and/or contract funding from Merck. The other authors declare no conflicts of interest.

## Data Availability

The data that support the findings of this study are available from NCI SEER‐Medicare. Restrictions apply to the availability of these data, which were used under license for this study. Data are available from the author(s) with the permission of NCI SEER‐Medicare.
